# Supplementary material for: Sp100A isoform promotes HIRA histone chaperone localization to PML nuclear bodies
Source: bioRxiv. 2025 Mar 6:2025.03.06.641437. Preprint. [Version 1] doi: 10.1101/2025.03.06.641437 (PMC12190398; doi:10.1101/2025.03.06.641437)

Supplementary Table 1: Genotype of Sp100 CRISPR knockout cells

	**Exon 3**	**Exon 6**	**Exon 8**
**CRISPR guide**	1	2	3, 4, 5
**Vector 1 #9**	wildtype	wildtype	wildtype
**Vector 2 #10**	wildtype	wildtype	wildtype
**Sp100 KO 1 #11**	Wildtype, 15bp del1, 15bp del2	wildtype	35bp del1, 35bp del2
**Sp100 KO 2 #12**	Wildtype, 11bp del 16bp del	wildtype	35bp del1, 35bp del2
**Sp100 KO 3 #13**	Wildtype, 15bp del1, 15bp del2	wildtype	35bp del1, 35bp del2
**Sp100 KO 4 #28**	158bp 28S rRNA insertion	wildtype	35bp del, 36bp del
**Sp100 KO 5 #33**	158bp 28S rRNA insertion	wildtype	36bp del

Supplemental figure 1

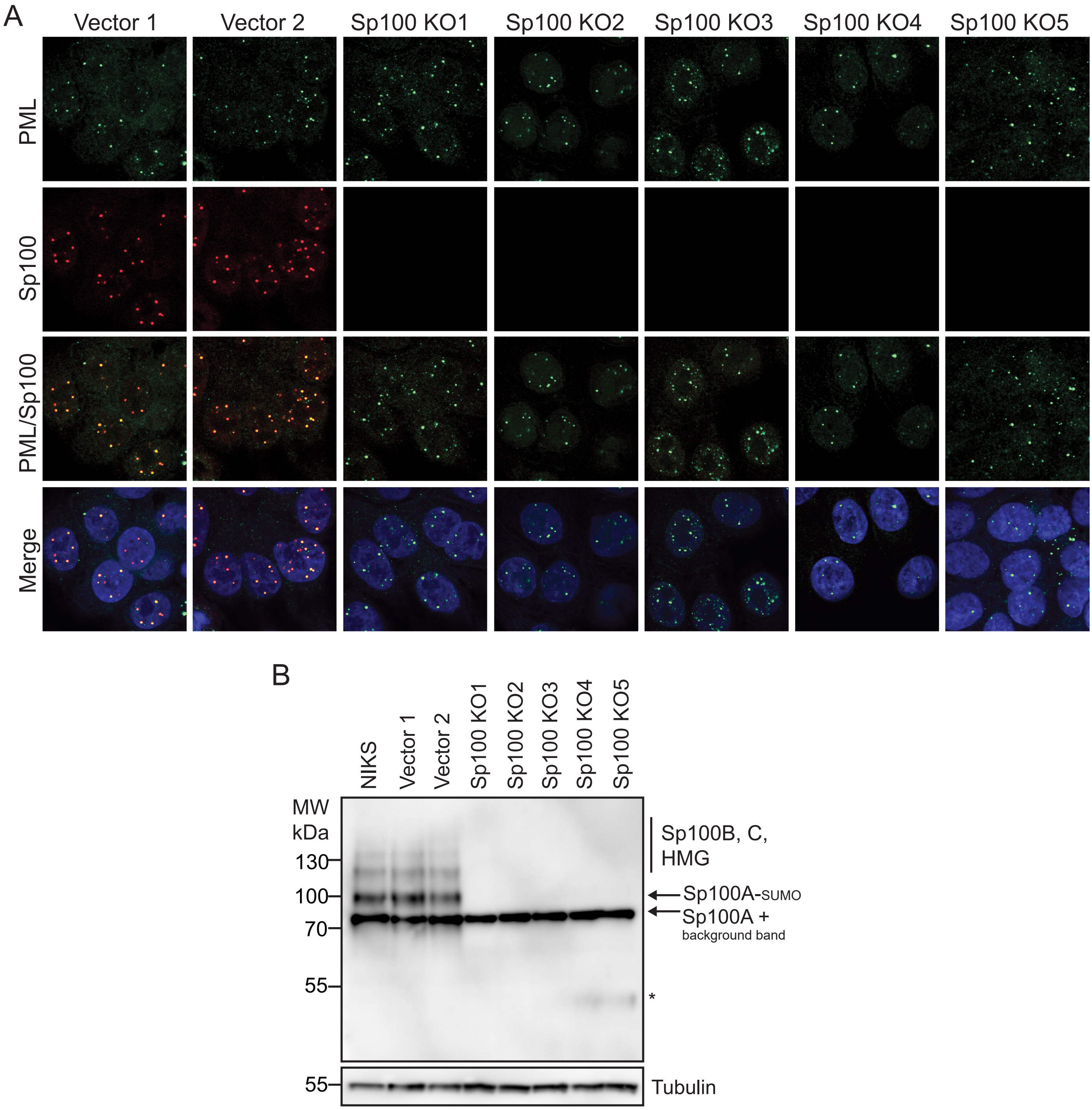

Supplemental figure 2

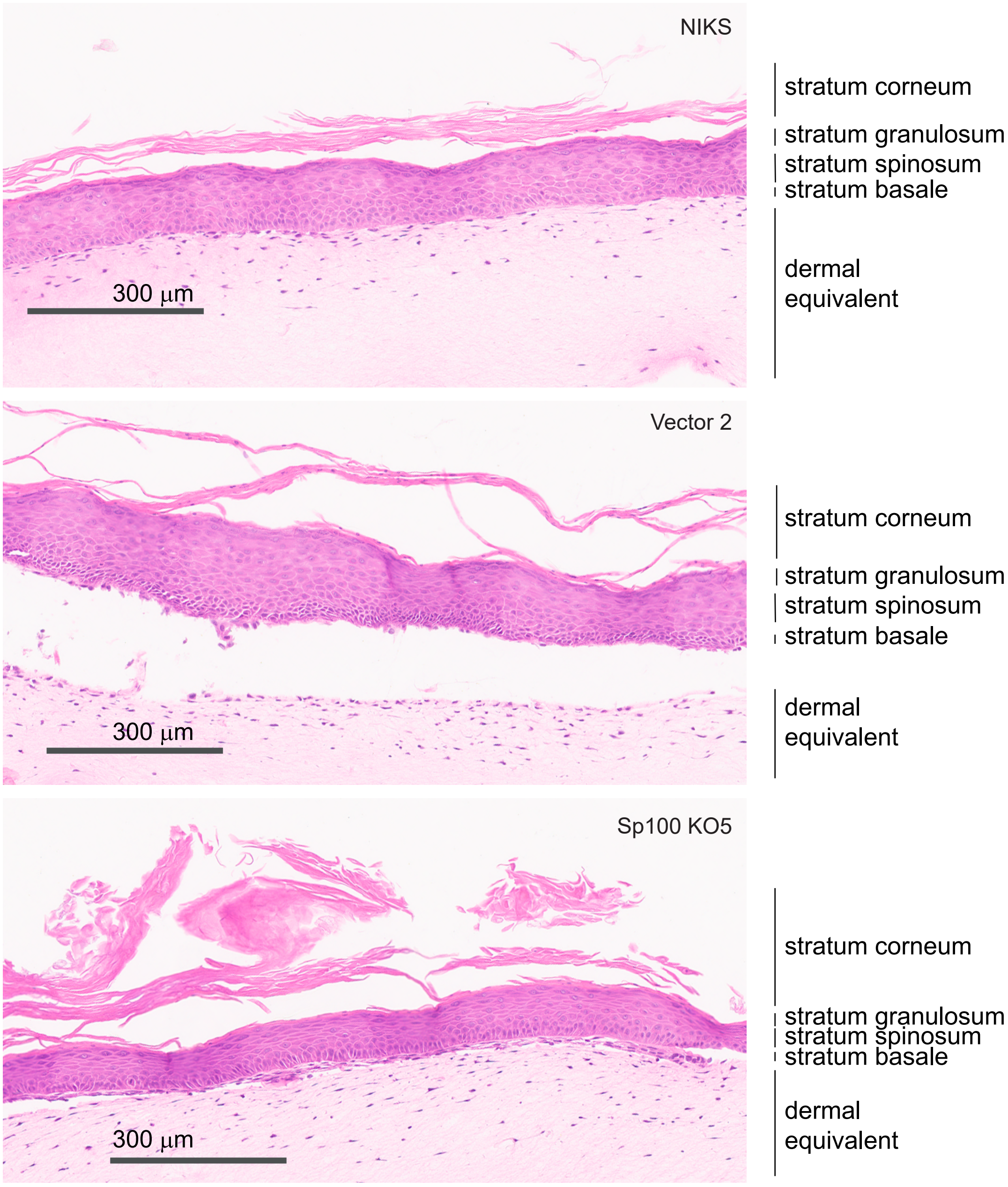

Supplemental figure 3

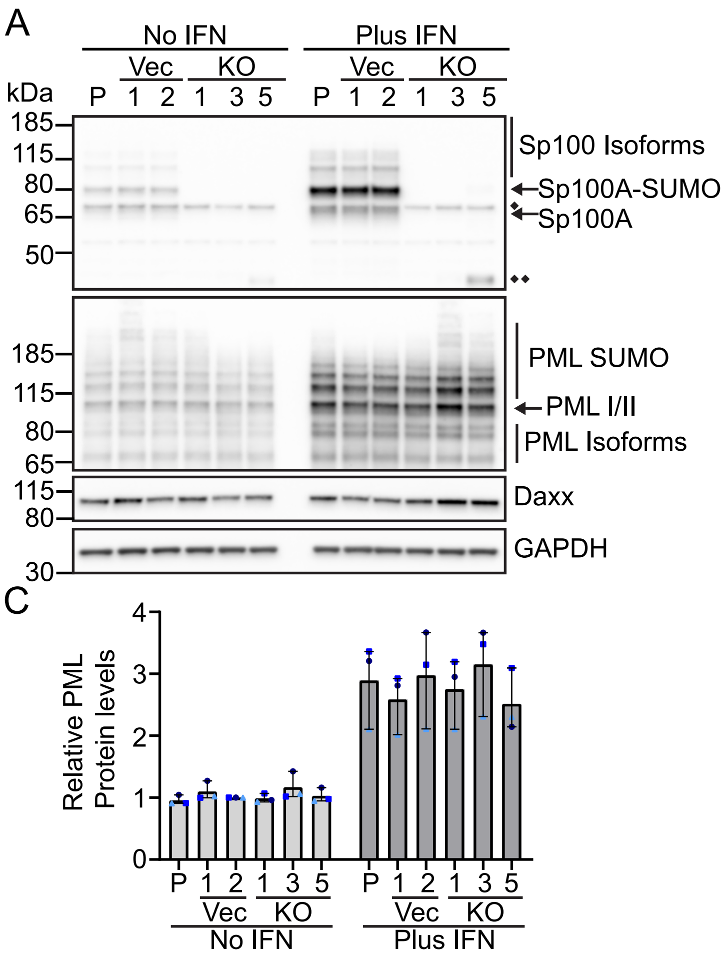

Supplemental figure 4

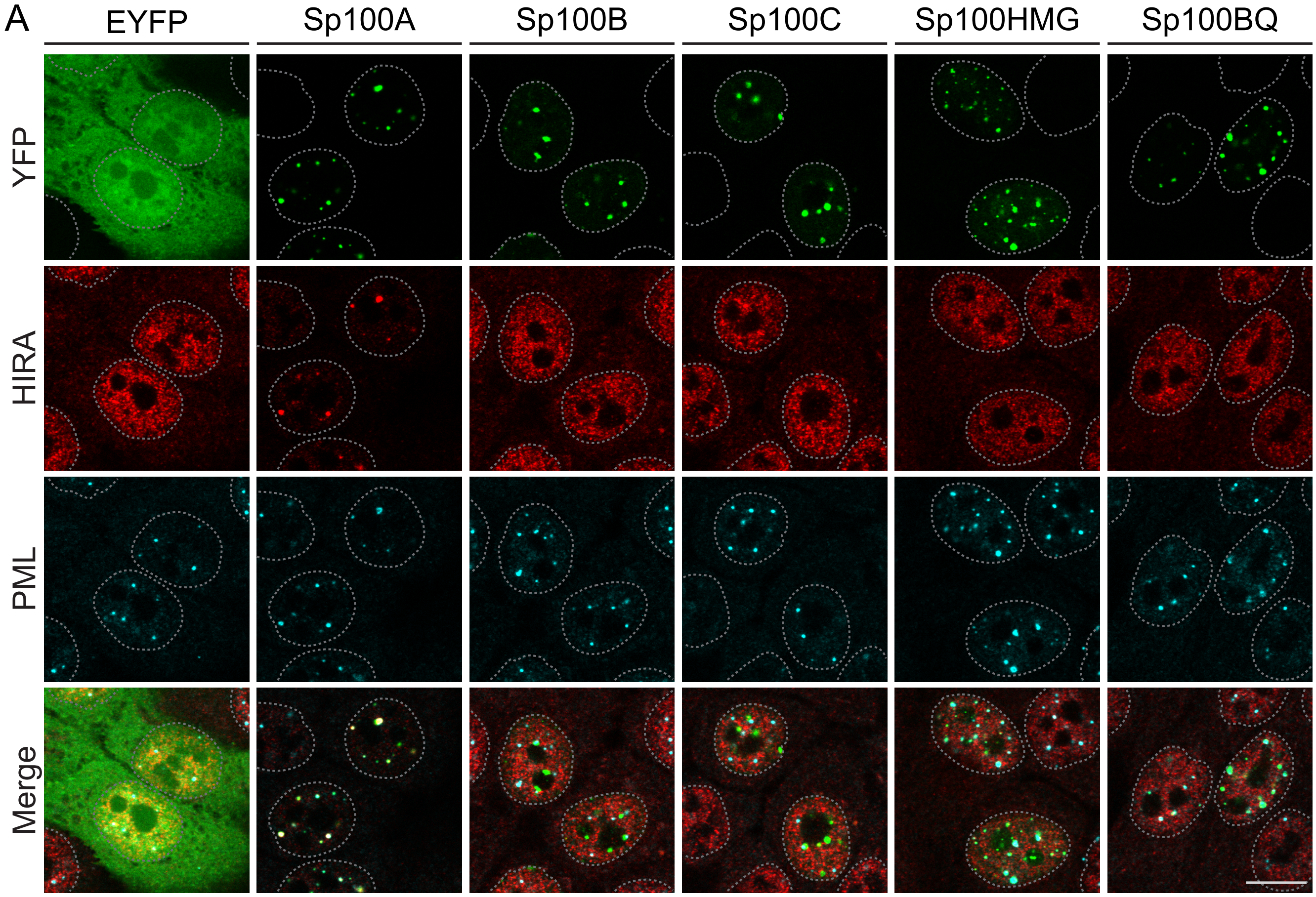

Supplemental figure 5

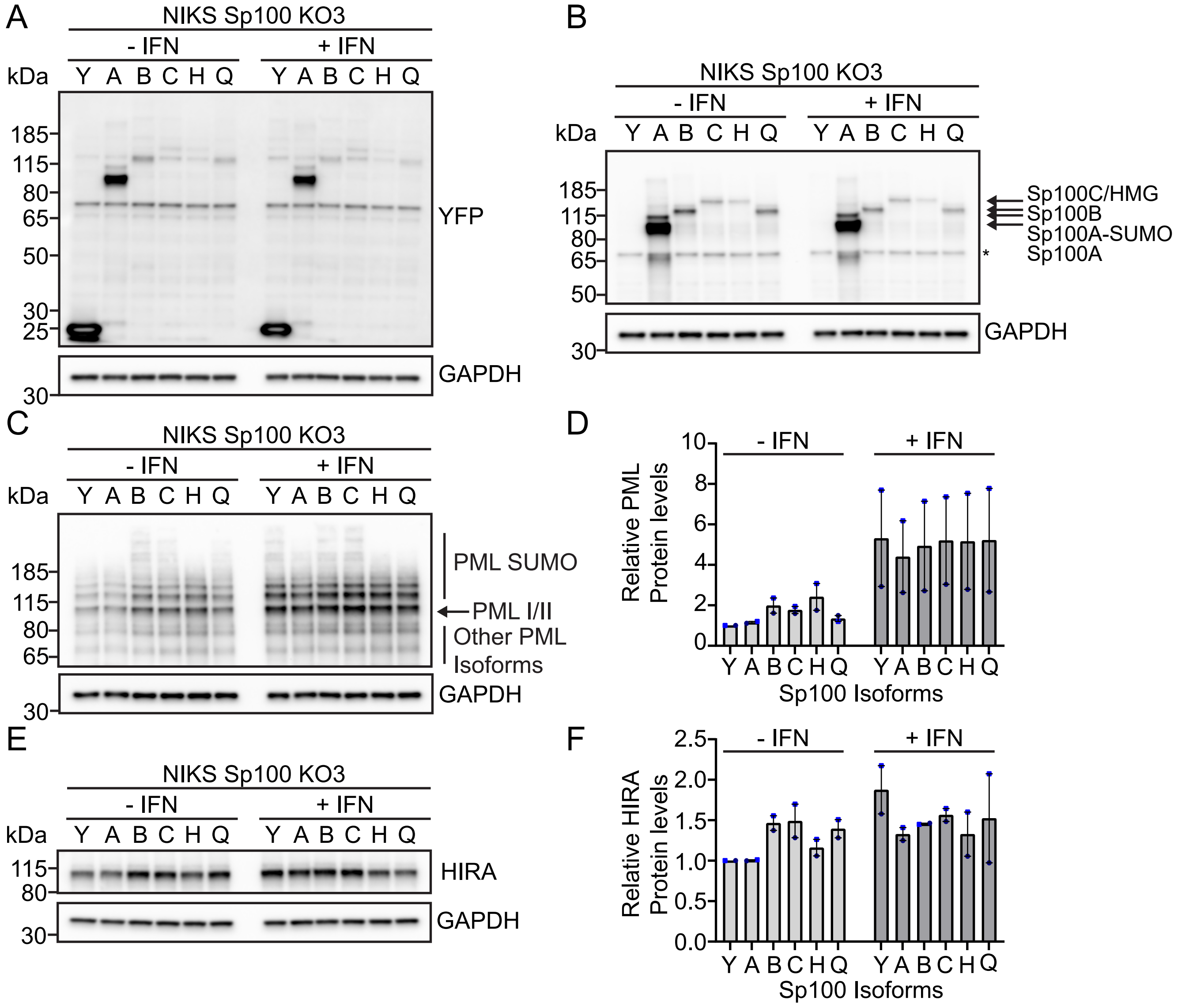

Supplemental figure 6

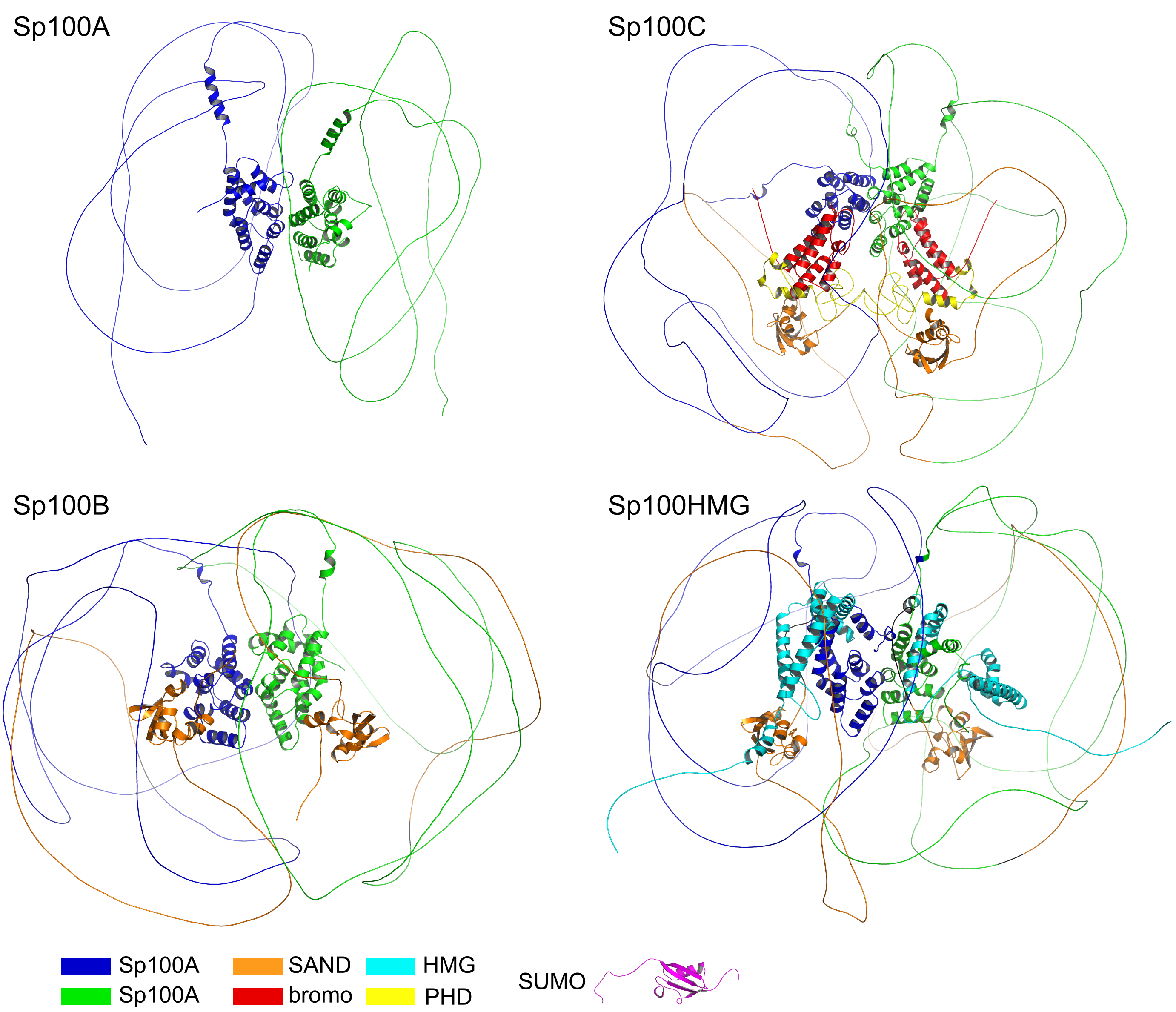

Supplement: Supplement 1 — Supplementary Figure 1 Screening knockout clones A. Identification of Sp100 KO cells by immunofluorescent staining for PML and Sp100. Sp100 KO cell pools were screened by the absence of Sp100 staining in PML nuclear bodies. Shown are two Vector wildtype control and five Sp100 KO cell pools that were selected for further analysis. B. Confirmation that Sp100 KO cells do not express Sp100 isoforms by Western blot analysis Immunoblot analysis of protein lysates from NIKS, the two Vector wildtype control and the five Sp100 KO cell pools shown in Supplementary Figure 1. Note: a novel truncated Sp100 product is detected in Sp100 KO4 and 5 and is indicated by an *. Supplementary Figure 2. Sp100 depletion does not affect keratinocyte differentiation. Sp100 depletion does not affect formation of a stratified epithelium in organotypic rafts. Representative organotypic rafts generated from NIKS, Vector 2 and KO5 cells and stained with H&E. Scale bar represents 300 μm. Supplementary Figure 3 PML and Daxx protein levels are unaltered in Sp100 KO keratinocytes. These experiments are controls for those in Figure 2, analyzing Sp100, Daxx and PML at PML-NBs. A. Immunoblots of NIKS Parental, Vector clones 1 and 2, and Sp100 KO clones 1, 3, and 5) cells treated with or without IFNα for 48 hours. Protein levels of Sp100, PML, Daxx, and GAPDH were detected as described in Materials and Methods. The single diamond symbol indicates a non-specific band, and the double diamond symbol denotes a truncated Sp100 polypeptide. The blots are representative of three independent experiments. B. Quantitation of panel A, showing the levels of total Sp100 protein normalized to GAPDH levels. Levels are shown relative to NIKS Vector 2 minus IFN (n=3). C. Quantitation of panel A, showing the total PML protein levels normalized to GAPDH. Data are shown relative to NIKS Vector 2 minus IFN value. D. Quantitation of panel A, showing the protein levels of Daxx normalized to GAPDH and relative to NIKS Vector 2 [file media-1.pdf]
